# Arbuscular mycorrhizal fungi associations of vascular plants confined to river valleys: towards understanding the river corridor plant distribution

**DOI:** 10.1007/s10265-014-0680-9

**Published:** 2014-11-25

**Authors:** Agnieszka Nobis, Janusz Błaszkowski, Szymon Zubek

**Affiliations:** 1 Department of Plant Taxonomy, Phytogeography and Herbarium, Institute of Botany, Jagiellonian University, Kopernika 27, 31-501 Kraków, Poland; 2 Department of Plant Protection, West Pomeranian University of Technology, Słowackiego 17, 71-434 Szczecin, Poland

**Keywords:** Arbuscular mycorrhizal fungi (AMF), Central Europe, Dark septate endophytes (DSE), Linear distribution pattern of vascular plants, *Olpidium* spp., Plant geography

## Abstract

The group of river corridor plants (RCP) includes vascular plant species which grow mainly or exclusively in the valleys of large rivers. Despite the long recognized fact that some plant species display a corridor-like distribution pattern in Central Europe, there is still no exhaustive explanation of the mechanisms generating this peculiar distribution. The main goal of this study was therefore to investigate whether arbuscular mycorrhizal fungi (AMF) and fungal root endophytes influence the RCP distribution. Arbuscular mycorrhizae (AM) were observed in 19 out of 33 studied RCP. Dark septate endophytes (DSE) and *Olpidium* spp. were recorded with low abundance in 15 and 10 plant species, respectively. The spores of AMF were found only in 32 % of trap cultures established from the soils collected in the river corridor habitats. In total, six widespread AMF species were identified. Because the percentage of non-mycorrhizal species in the group of RCP is significant and the sites in river corridors are characterized by low AMF species diversity, RCP can be outcompeted outside river valleys by the widespread species that are able to benefit from AM associations in more stable plant-AMF communities in non-river habitats.

**Electronic supplementary material:**

The online version of this article (doi:10.1007/s10265-014-0680-9) contains supplementary material, which is available to authorized users.

## Introduction

As early as in the second half of the nineteenth century German phytogeographers noticed that some vascular plant species demonstrate specific linear distribution pattern reflecting their confinement to river valleys (e.g. Ascherson 1859; Loew 1879). Burkart (2001) proposed the English term ‘river corridor species’ for this group of plants and presented the first preliminary list of 129 species growing mainly or exclusively in the corridors of large rivers in Central Europe. The list was recently supplemented by Nobis and Skórka (2014). Confinement of the particular species to river corridors can be estimated by calculation of its river corridor-specificity index (RCSI). The index calculation procedure is included in the works by Fischer et al. (2010) and Nobis and Skórka (2014).

Different hypotheses have been proposed to explain the phenomenon in question. Ascherson (1859) and Loew (1879) supposed that in the case of river corridor plants (RCP) water is the most important vector of their propagules. Therefore, they believed that hydrohory explains their linear distribution pattern. In fact, there is little evidence supporting this hypothesis (Andersson and Nilsson 2002; Nilsson et al. 2010; Nobis and Skórka 2014). A second probable explanation of the unique distribution pattern is typical of floodplain ecosystems disturbance by water resulting in a complete or partial reduction in plant cover (Jentsch and Seitz 1997; Vent and Benkert 1984). The published data confirm significance of disturbance in corridor-like distribution of some alien and native vascular plant species (Andres and Westhus 2000; Burkart 1995; Pyšek and Prach 1994). According to the climatic hypothesis, the occurrence of warmer summer conditions in river valleys than in their surroundings is an important factor for RCP distribution (Müller-Stoll et al. 1962; Vent and Benkert 1984). Finally, differences in nutrient supply in floodplains and in their surrounding areas suggest that RCP may require high nutrition levels (Fischer 1996; Vent and Benkert 1984). According to Nobis and Skórka (2014), who analyzed ecological features of RCP using the Ellenberg’s system of indicator values, corridor species differ from widespread species (i.e. species which do not show confinement to river corridors and are very frequent in the area of Poland) in several traits, with requirements for a higher temperature and alkalinity being the most distinctive features of the former. However, no significant difference in the nutrient requirements between the two groups of plants has been observed.

River corridor distribution patterns are not only affected by different abiotic environmental factors. Propagule transport by birds migrating along the river valleys turned out to be important for some RCP (e.g. Gams 1927). Moreover, Hensgen et al. (2011) indicated that the distribution of some RCP may be influenced by gastropods. It cannot be excluded that also other groups of organisms can influence the plants confined to river valleys. Among soil microorganisms, arbuscular mycorrhizal fungi (AMF) play a key role in the impact on plant performance. They have been found to influence the growth, vitality, reproduction and survival rate of plants as well as determine plant community structure (Perotto et al. 2013; Smith and Read 2008). As no studies focused on mycorrhiza associations of the group of plants in question have been performed so far, the aim of the present study was therefore to investigate their arbuscular mycorrhiza (AM) status, the degree of root colonization by AMF and to evaluate AMF species diversity in soils surrounding roots of plants demonstrating corridor-like distribution in Poland.

Winter, spring and early summer floods typical of river corridors may result not only in a complete or partial reduction in plant cover, but can also affect soil microorganism consortia, including AMF. On the other hand, summer in the river valleys is notably dry, so the microorganisms inhabiting river corridor soils are influenced by very variable soil moisture. We therefore expected that plant species confined to river corridors form arbuscular mycorrhiza (AM) rarely or not at all, or the degree of colonization of their roots is low due to the lack or low number of AMF species and their propagules in river corridor soils. We put forward the hypothesis that RCP are consequently outcompeted outside river valleys by the widespread species that are able to benefit from AM associations in more stable plant-AMF communities in non-river habitats.

## Materials and methods

### Study area and material sampling

The material was collected in 2011 and 2012 in the lower course of the San River Valley (Fig. S1). San River is situated in southeastern Poland and western Ukraine. It is a right-bank tributary of the Vistula River. With its length of 443 km it is the 6th-longest Polish river. Lower San River Valley comprises many natural and semi-natural ecosystems. It is therefore considered as very valuable for biodiversity conservation. A significant part of the Lower San River Valley is protected as a Special Area of Conservation (SACs) established under NATURA 2000. In the study area the following habitats can be found: muddy river banks, watercourse veils, reed beds, riverine willow scrub, aluvial forests, meadows, dry grasslands, oxbow lakes, herbaceous fringes, pastures and arable fields. Many of the listed habitats are suitable for vascular plants which are included in the list of RCP of Poland proposed by Nobis and Skórka (2014).

Altogether, 33 RCP species from 100 randomly selected locations (3, rarely 2 or 4 sites per species) were collected during flowering or early seed formation period mostly from typical habitats. The roots of each collected specimen were excavated with surrounding soil. The root systems, or their fragments, were then placed in plastic containers holding 50 % ethanol in water. The soil samples were dried at room temperature of around 22 °C. The list of all studied species together with the data on the name and coordinates of localities as well as the habitats where their specimens were collected are presented in the supplementary material (Table S1). The nomenclature of vascular plant species follows Mirek et al. (2002).

### Root staining and the assessment of fungal colonization

The roots were prepared in accordance with the modified Phillips and Hayman (1970) method. They were cleared in 10 % KOH for 24 h and then rinsed in water. The material was then acidified in 5 % lactic acid in water (24 h), stained with 0.05 % aniline blue in 80 % lactic acid (72 h), and finally stored in 80 % lactic acid until analyzed. Root fragments approximately 1 cm long, at 30 fragments per one repetition sample, were mounted on slides in glycerol:lactic acid (1:1) and pressed using coverslides.

Fungal root colonization was assessed by means of a Nikon Eclipse 80i light microscope with differential interference contrast (DIC). AMF colonization and morphology were identified on the basis of hyphae growing (a) intercellularly, forming arbuscules terminally in cortical cells (the *Arum*-type of AM morphology); (b) intracellularly with arbuscules developed on coils in cortical cells (*Paris*-type); or (c) forming intermediate types (Dickson 2004). The method proposed by Trouvelot et al. (1986) was followed for the assessment of the degree of AM development. The parameters evaluated were mycorrhizal frequency, relative mycorrhizal root length, and relative arbuscular richness. An estimate of the mycorrhizal frequency (F_AMF _%) is given as the ratio between root fragments colonized by AMF mycelium and the total number of root fragments analysed. The relative mycorrhizal root length (M_AMF _%) is an estimate of the amount of root cortex colonized by AMF relative to the entire root system investigated. The relative arbuscular richness (A_AMF _%) is an estimate of arbuscule richness in the entire root system analysed (Trouvelot et al. 1986).

During the assessment of AMF colonization, we also observed fungal endophytes that accompanied AMF in roots, namely dark septate endophytes (DSE) and fungi from the genus *Olpidium*. DSE colonization was identified on the basis of regularly septate hyphae, usually dark pigmented, with facultatively occurring sclerotia (Jumpponen 2001). Additionally, the frequency of occurrence of *Olpidium* sporangia (Webster and Weber 2007) was assessed. In the case of DSE and *Olpidium* colonization, the frequency of the occurrence of the structures of these fungi in roots (F_DSE _%, F_Olp _%) was estimated as detailed above for AMF.

Because parameters specifying the degree of AM development (F_AMF _%, M_AMF _% and A_AMF _%) as well as the frequency of the occurrence of fungal root endophytes (F_DSE _% and F_Olp _%) and river corridor-specificity indexes (RCSI) did not meet the assumptions of normality, non-parametric tests were applied in statistical analyses. The relationships between root colonization of RCP species by AMF, DSE or *Olpidium* spp. and the river corridor-specificity index were analysed by means of Spearman’s rank correlation coefficients. The statistical calculations were performed using the STATISTICA 10 software package.

### Establishment of the AMF trap cultures

The soils excavated from under each plant species collected in 2012 were used to establish AMF trap cultures. For the establishment of each trap culture, 100 g of air-dried soil was placed in 9 cm × 12.5 cm, 500 ml, plastic pots containing autoclaved, commercially available, coarse-grained sand. *Plantago lanceolata* was used as the host plant. In total, 56 trap cultures were established.

### AMF spore isolation and identification

Ten months after establishment of the trap cultures, AMF spores were extracted using the wet sieving and decanting method of Gerdemann and Nicolson (1963). AMF species were identified according to Błaszkowski (2012). The shape and size of spores were determined from intact spores mounted in a drop of water or lactic acid on a microscope slide. Dimensions were determined using a compound microscope equipped with an ocular micrometer. The thickness of layers of a spore wall and inner walls was measured in material mounted on a slide in a drop of polyvinyl alcohol/lactic acid/glycerol (PVLG) and in a mixture of PVLG/Melzer’s reagent (4:1, v/v) (Omar et al. 1979), and observed under a compound microscope equipped with micrometer eyepiece. Spore colour was determined in water and observed under a dissecting microscope. Colour of spore wall layers and inner wall layers was determined in spores crushed in PVLG, and in PVLG/Melzer’s reagent when staining reactions were examined. Colours were determined according to Kornerup and Wanscher (1983). The observations of AMF spore features were performed using an Olympus BX51 light microscope with differential interference contrast (DIC). The fungal species and family names given in Table 1 are after Schüßler and Walker (2010), apart from *Paraglomus majewskii* and *Septoglomus constrictum*, which follows Błaszkowski et al. (2012) and Redecker et al. (2013), respectively.

**Table 1:** Arbuscular mycorrhizal fungi (AMF, Glomeromycota) isolated from the trap cultures established with the soils collected from the selected stations of river corridor plant species

Fungal family	Fungal species	River corridor plant species^a^
Claroideoglomeraceae	*Claroideoglomus claroideum*	*Alisma lanceolatum* ^4^
		*Myosotis sparsiflora* ^41^
	*Claroideoglomus drummondii*	*Aethusa cynapioides* ^2^
		*Gratiola officinalis* ^28^
		*Potentilla supina* ^45^
		*Roripa austriaca* ^49,^ ^50^
Diversisporaceae	*Diversispora* sp.	*Gratiola officinalis* ^28^
Glomeraceae	*Septoglomus constrictum*	*Butomus umbelatus* ^8, 9^
		*Cirsium canum* ^18^
		*Myosotis sparsiflora* ^40^
		*Petasites spurius* ^44^
		*Roripa austriaca* ^50^
		*Viola stagnina* ^55,^ ^56^
	*Funneliformis mosseae*	*Butomus umbelatus* ^10^
	*Rhizophagus irregularis*	*Aethusa cynapioides* ^3^
Paraglomeraceae	*Paraglomus majewskii*	*Potentilla supina* ^46, 47^
		*Roripa austriaca* ^49^

The superscript number indicates the location of isolation as listed in Table S1

^a^ The name of plant species from which an AMF trap culture was isolated

### Chemical soil analyses

Soil samples collected from under studied RCP species were air-dried and sieved (<2 mm). All the samples were analyzed for pH in aqueous solution. The total nitrogen (N %) in soil samples was determined by means of the Kjeldahl method, and the organic carbon (C %) using the Tiurin method (Mocek and Drzymała 2010). The assessment of plant-available phosphorus (P_2_O_5_) and potassium (K_2_O) contents were done after extraction with ammonium lactate acetic acid according to Egner et al. (1960). Exchangeable cations (K^+^, Na^+^, Mg^2+^ and Ca^2+^) were measured using a flame photometer and spectrophotometer in 1 N ammonium acetate.

Correlations between root colonization of RCP species by AMF, DSE and *Olpidium* spp. and particular site parameters were analysed using non-parametric Spearman rank correlation.

## Results

### Fungal root colonization of RCP species

Arbuscular mycorrhizae (AM) were observed in 19 out of the 33 studied RCP species. The mean values of mycorrhizal frequency parameter (F_AMF _%) ranged from 37.7 % in *Lindernia procumbens* to 100 % in *Allium* *scorodoprasum*. In the case of relative mycorrhizal root length (M_AMF _%), the mean values were lowest in *Senecio fluviatilis* (8.7 %) and highest in *Allium scorodoprasum* (87.3 %). Similar trends were also found in relative arbuscular richness (A_AMF _%). The mean values of this parameter varied from 8.5 % in *Senecio fluviatilis* to 85.8 % in *Allium scorodoprasum*. Mycorrhizae of 12 plant species were of the *Arum* morphology, five taxa showed *Paris*-type of AM colonization, and both *Arum* and *Paris* morphology in the same root systems was found in two species (Table 2).

**Table 2:** Root colonization of river corridor plant species by arbuscular mycorrhizal fungi (AMF), dark septate endophytes (DSE) and *Olpidium* spp.

Family	Plant species	RCSI^a^	Previous reports on AM status^b^	AM type^c^	AMF ^d^			Fungal endophytes^e^	
					F	M	A	F_DSE_	F_Olp_
Alliaceae	*Allium angulosum*	0.86	**S**-NS; **G**/**F**-AM^1^, (NM^1^), A^2, 4^	A	97.1 ± 5.1	73.8 ± 14.7	73.2 ± 15.3	58.4 ± 11.2	–
	*Allium scorodoprasum*	0.82	**S**-AM^1^	A	100	87.3 ± 3.4	85.8 ± 4.8	37.5 ± 17.0	–
Alismataceae	*Alisma lanceolatum*	0.81	**S/G**-AM, NM^1^; **F**-AM, NM^1^, A^2^	NM	–	–	–	–	–
Apiaceae	*Aethusa cynapioides*	0.76	**S**-NS; **G**-AM^1^; **F**-AM^1, 6^, (NM^1^), A^2^, P^2, 4, 7, 9^, I^2, 9, 11^	P	73.4 ± 26.9	35.6 ± 16.8	34.9 ± 17.0	–	–
	*Chaerophyllum bulbosum*	0.66	**S**-NS; **G**-AM, NM^1^	P	59.3 ± 22.3	36.1 ± 14.1	35.9 ± 14.0	–	8.7 ± 17.5
	*Cnidum dubium*	0.77	**S/G**-NS	P	82.9 ± 24.4	54.9 ± 21.2	65.8 ± 27.9	2.1 ± 3.6	14.8 ± 18.1
	*Eryngium planum*	0.73	**S**-NS; **G**-AM^1^, (NM^1^), A^2^, P^2^	P	82.5 ± 30.4	46.8 ± 30.6	46.8 ± 30.6	–	6.1 ± 10.5
Asteraceae	*Cirsium canum*	0.73	**S**-NS; **G**-AM^1^, (NM^1^), A^2^; **F**-AM^1, 6^, (NM^1^), A^2, 4, 5, 7, 8, 9, 10, 11^, (P^2^, NM^11^)	A	75.4 ± 6.9	34.5 ± 17.1	28.1 ± 19.9	13.81 ± 10.2	1.1 ± 1.9
	*Petasites spurius*	0.86	**S**-NS; **G**-AM, NM^1^	A	86.2 ± 10.5	41.4 ± 11.2	40.3 ± 11.3	15.9 ± 5.8	–
	*Senecio fluviatilis*	0.89	**S**-NS; **G**-AM^1^, (NM^1^), A^2, 4, 8, 10^	A	47.1 ± 35.8	8.7 ± 10.1	8.5 ± 10.0	–	–
Boraginaceae	*Lithospermum officinale*	0.83	**S**-NM^1^; **G**-AM^1^, NM ^1^; **F**-AM^1^, NM^1^, A^2, 8, 9^, P^2^	A	82.5 ± 4.4	46.7 ± 19.8	44.0 ± 23.6	–	–
	*Myosotis sparsiflora*	0.73	**S**-NS; **G**-AM^1^, NM^1^	NM	–	–	–	–	70.0 ± 18.8
Brassicaceae	*Barbarea stricta*	0.76	**S**-NS; **G**-NM^1^; **F**-NM^1, 4, 5, 8, 10, 11^, (AM^1^, I^4^)	NM	–	–	–	4.9 ± 5.9	–
	*Rorippa austriaca*	0.80	**S**-NS; **G**-NM^1^, (AM^1^)	NM	–	–	–	6.7 ± 6.7	–
	*Sisymbrium strictissimum*	0.93	**S**-NS; **G**-NM^1^, (AM^1^, I^4^)	NM	–	–	–	–	41.1 ± 19.2
Butomaceae	*Butomus umbelatus*	0.69	**S/G/F**-NM^1^	AP	38.2 ± 51.4	19.6 ± 31.7	19.6 ± 31.7	2.9 ± 5.0	–
Caryophyllaceae	*Cucubalus baccifer*	0.76	**S/G**-NS; **F**-AM^1^, NM^1, 7, 8^, (A^2, 4^)	NM	–	–	–	4.8 ± 8.2	8.9 ± 7.7
Chenopodiaceae	*Chenopodium ficifolium*	0.83	**S**-NS; **G/F**-NM^1, 4^, (AM^1^)	NM	–	–	–	–	7.0 ± 8.4
	*Kochia laniflora*	0.87	**S/G**-NS	NM	–	–	–	–	–
Cyperaceae	*Bolboschoenus maritimus*	0.78	**S/G**-AM^1^; **F**-AM^1^, NM^1^, (A^2^, P^2^)	NM	–	–	–	–	4.5 ± 5.2
	*Carex praecox*	0.73	**S**-NS; **G**-AM^1, 6^, NM^1, 6^	NM	–	–	–	3.3 ± 5.8	–
	*Cyperus fusus*	0.68	**S**-NS; **G**-AM^1^, NM^1^	NM	–	–	–	–	–
Dipsacaceae	*Dipsacus laciniatus*	0.91	**S**-NS; **G**-AM^1^, NM^1^; **F**-AM^1^, (NM^1^), A^2, 8^	A	95.5 ± 1.6	64.3 ± 3.4	58.6 ± 6.8	5.0 ± 7.1	–
Equisetaceae	*Equisetum ramosissimum*	0.90	**S/G/F**- AM^1, 6^, NM^1^, P^2^	NM	–	–	–	–	–
Lamiaceae	*Chaiturus marrubiastrum*	0.88	**S/G**-NS; **F**-AM^1, 6^, (NM^1^), A^2, 4, 5, 7, 8, 9, 10, 11^, (P^2, 7^, I^2^)	A	60.2 ± 27.6	30.2 ± 16.6	22.8 ± 9.6	3.3 ± 3.3	1.1 ± 1.8
	*Scutellaria hastifolia*	0.90	**S**-NS; **G**-AM^1^, NM^1^, A^10^	A	81.0 ± 12.5	44.1 ± 13.9	34.9 ± 12.3	3.4 ± 3.3	–
Malvaceae	*Lavatera thuringiaca*	0.69	**S/G**-NS; **F**-AM^1^, (NM^1^); A^2, 5, 7^, P^2, 5, 7^, I^2^	AP	96.7 ± 5.8	58.4 ± 20.0	56.7 ± 22.6	–	–
Poaceae	*Leymus arenarius*	0.71	**S**-NS; **G**-AM^1^, NM^1^; **F-**AM^1, 6^, (NM^1, 3, 6^), A^2, 3, 5^, P^2, 3, 4, 5, 8^, I^2, 3, 4, 5^	NM	–	–	–	–	–
Rosaceae	*Potentilla supina*	0.69	**S**-NS; **G**-AM^1^, (NM^1^), A^9^; **F-** AM^1, 6^, (NM^1^), A^2, 4, 8, 9, 10, 11^, (P^2^)	A	85.0 ± 3.0	46.4 ± 13.7	42.9 ± 10.1	–	–
Scrophulariaceae	*Gratiola officinalis*	0.85	**S/G**-A^9^; **F**-AM^1, 6^, NM^1, 8, 9^, A^2, 4, 7, 9^, (I^2, 4^, P^2^)	A	90.5 ± 3.9	32.3 ± 0.1	26.7 ± 2.7	–	–
	*Limosella aquatica*	0.80	**S/G**-NS	NM	–	–	–	–	–
	*Lindernia procumbens*	0.87	**S/G**-NS	A	37.7 ± 31.0	17.6 ± 19.6	17.6 ± 19.6	4.4 ± 5.1	–
Violaceae	*Viola stagnina*	0.75	**S**-NS; **G/F**-AM^1, 6^, (NM^1^), P^2^, (A^2, 7, 10^)	P	76.2 ± 8.6	38.3 ± 14.3	35.9 ± 14.0	8.9 ± 7.7	–

^a^ River corridor-specificity indexes estimating confinement of the particular species to river corridors in Poland according to Nobis and Skórka (2014)

^b^ AM status and AM morphotype previously reported in the studied species (**S**), genera (**G**) and families (**F**) according to the checklists: ^1^ Wang and Qiu (2006), ^2^ Dickson et al. (2007), and the reports published thereafter or not included in the checklists: ^3^ Sathiyadash et al. (2010), ^4^ Shah et al. (2009), ^5^ Muthukumar and Prakash (2009), ^6^ Weishampel and Bedfor (2006), ^7^ Zubek and Błaszkowski (2009), ^8^ Zubek et al. (2008), ^9^ Zubek et al. (2011a), ^10^ Zubek et al. (2011b), ^11^ Zubek et al. (2012). *AM* arbuscular mycorrhiza reported without information on AM morphotype, *NM* non-mycorrhizal, *NS* not surveyed, *A* *Arum*-type, *P* *Paris*-type, *I* intermediate type. The information given in parenthesis indicates rarely observed AM colonization or AM morphotypes in the genera and families

^c^ AM status and morphotype (according to Dickson 2004) observed in this study. *A* *Arum*-type, *P* *Paris*-type, *AP* both *Arum*-type and *Paris*-type, *NM* non-mycorrhizal

^d^ The presence of arbuscular mycorrhizal fungi (AMF). Mycorrhizal parameters (percentages; mean ± SD): *F* mycorrhizal frequency, *M* relative mycorrhizal root length, *A* relative arbuscular richness

^e^ Frequency of the occurrence of fungal root endophytes (percentages; mean ± SD): *F*_*DSE*_ the mycelium of dark septate endophytes, *F*_*Olp*_ the sporangia of *Olpidium* spp.

No AMF mycelium was found in the roots of 14 plant species. All species studied from Alismataceae, Brassicaceae, Caryophyllaceae, Chenopodiaceae, Cyperaceae, Equisetaceae and Poaceae families were non-mycorrhizal. Moreover, *Myosotis sparsiflora* (Boraginaceae) and *Limosella aquatica* (Scrophulariaceae) were also found not to form AM symbiosis.

Dark septate endophytes (DSE) were found in 15 plant species, both in the roots colonized by AMF and devoid of AM. The mean frequency of DSE occurrence in roots (F_DSE _%) was low and ranged from 2.1 % in *Cnidum dubium* to 58.4 % in *Allium angulosum* (Table 2). The abundance of DSE in roots was low in all plant species. Only single hyphae, accompanied sporadically by sclerotia, were found in the rhizodermis and outer cortex.

The sporangia of *Olpidium* spp. were found in ten plant species. The frequency of occurrence of these structures (F_Olp _%) varied from 1.1 % in *Chaiturus marrubiastrum* and *Cirsium canum* to 70 % in *Myosotis sparsiflora* (Table 2). The abundance of sporangia in roots was low in all the plant species. Only single sporangia were found in the rhizodermis.

No significant correlation between root colonization by AMF or fungal endophytes and river corridor-specificity index of studied RCP was found (Table 3).

**Table 3:** Spearman’s rank correlation coefficients between root colonization of river corridor plant species by arbuscular mycorrhizal fungi/fungal endophytes and particular soil parameters/river corridor-specificity index

	pH	N (%)	C (%)	Organic matter (%)	C/N	K_2_O	P_2_O_5_	K^+^	Na^+^	Ca^+^	Mg^2+^	RCSI
F_AMF _%	−0.14	0.27	0.27	0.28	−0.08	0.02	−0.13	−0.03	0.16	0.23	0.20	−0.10
M_AMF _%	−0.16	0.34	0.32	0.34	−0.16	0.09	−0.07	0.04	0.25	0.31	0.27	−0.15
A_AMF _%	−0.16	0.33	0.32	0.33	−0.16	0.09	0.07	0.04	0.25	0.32	0.29	−0.18
F_DSE _%	−0.32	0.52*	0.54*	0.53*	−0.04	0.02	0.10	0.05	0.18	0.26	0.22	−0.25
F_Olp _%	0.37*	0.14	0.07	0.08	−0.22	0.36*	0.21	0.34	−0.01	0.23	0.37*	−0.20

For explanation of abbreviation, see Table 1 or the main text. Correlation coefficients greater than 0.5 are shown in bold

* Statistically significant (*P* ≤ 0.001)

### The presence of AMF in the river corridor soils

The spores of AMF were only found in 18 out of 56 trap cultures established from the soils collected in the river corridor habitats. In 15 trap cultures, a single fungal species was found, and three cultures contained two taxa. In total, the spores of six AMF species were isolated. In addition, one spore morphotype with glomoid spores similar to those of *Diversispora* spp. was found. The spores of *Septoglomus constrictum* were most frequently isolated and were present in eight cultures. In contrast, *Funneliformis mosseae*, *Diversispora* sp. and *Rhizophagus irregularis* were found in single cultures (Table 1).

### Soil chemical properties

The comparison of means and standard errors of means of soil chemical properties revealed differences between soils typical of particular species or groups of species representing certain types of habitat (Table 4). In some cases, considerable differences in soil parameters between stations of a given species were also observed.

**Table 4:** The chemical properties of the soil (mean ± SD) collected from under studied river corridor plant species

Plant species	pH	N (%)	C (%)	Organic matter (%)	C/N	Total content (mg per 100 g of dry soil)		Exchangeable cation (mg per 100 g of dry soil)			
						K_2_O	P_2_O_5_	K^+^	Na^+^	Ca^2+^	Mg^2+^
*Aethusa cynapioides*	7.5 ± 0.1	0.05 ± 0.02	0.7 ± 0.2	1.2 ± 0.3	14.4 ± 1.9	10.5 ± 4.2	8.7 ± 3.7	10.3 ± 2.6	13.0 ± 2.8	593.3 ± 208.4	3.8 ± 0.3
*Alisma lanceolatum*	6.5 ± 0.9	0.22 ± 0.06	2.5 ± 0.4	4.4 ± 0.7	11.8 ± 1.6	12.7 ± 9.4	9.8 ± 8.2	16.6 ± 6.7	8.7 ± 4.4	329.3 ± 78.7	11.8 ± 7.0
*Allium angulosum*	7.0 ± 1.0	0.28 ± 0.13	3.4 ± 1.4	5.9 ± 2.5	12.5 ± 2.6	14.4 ± 4.9	9.8 ± 3.3	13.4 ± 6.2	9.2 ± 2.4	766.7 ± 765.3	7.5 ± 3.1
*Allium scorodoprasum*	7.3 ± 0.2	0.36 ± 0.10	3.8 ± 1.1	6.5 ± 2.0	10.6 ± 0.3	43.0 ± 14.8	15.2 ± 4.2	32.7 ± 7.7	8.8 ± 0.9	492.0 ± 34.6	13.1 ± 0.2
*Barbarea stricta*	6.9 ± 0.9	0.18 ± 0.10	2.5 ± 1.7	4.3 ± 2.8	13.9 ± 3.0	16.9 ± 11.4	16.9 ± 10.7	14.0 ± 10.2	10.8 ± 5.3	320.7 ± 239.8	5.0 ± 6.1
*Bolboschoenus maritimus*	7.5 ± 0.5	0.09 ± 0.04	1.3 ± 0.7	2.2 ± 1.1	14.0 ± 4.5	10.4 ± 5.9	8.5 ± 4.4	9.3 ± 4.5	6.5 ± 0.7	270.0 ± 16.0	9.7 ± 2.6
*Butomus umbelatus*	6.4 ± 0.8	0.20 ± 0.07	2.7 ± 0.8	4.6 ± 1.4	13.7 ± 1.2	23.4 ± 7.7	15.9 ± 1.9	20.5 ± 6.7	15.5 ± 3.0	428.3 ± 46.5	10.8 ± 2.0
*Carex praecox*	6.6 ± 1.2	0.12 ± 0.06	1.9 ± 1.2	3.2 ± 2.1	15.3 ± 1.9	10.0 ± 10.7	10.3 ± 9.3	7.3 ± 6.9	5.4 ± 3.1	98.5 ± 91.4	1.6 ± 0.3
*Chaerophyllum bulbosum*	7.7 ± 0.1	0.17 ± 0.05	2.1 ± 0.5	3.7 ± 0.8	12.7 ± 0.8	33.7 ± 7.8	11.5 ± 1.1	26.0 ± 3.5	8.7 ± 1.1	484.5 ± 79.8	10.9 ± 3.5
*Chaiturus marrubiastrum*	6.8 ± 0.6	0.23 ± 0.10	2.9 ± 1.3	5.1 ± 2.2	12.6 ± 0.5	21.3 ± 4.1	18.7 ± 5.7	19.2 ± 3.8	10.0 ± 2.3	430.0 ± 112.7	12.2 ± 5.3
*Chenopodium ficifolium*	7.6 ± 0.2	0.11 ± 0.03	1.1 ± 0.3	1.9 ± 0.6	10.7 ± 0.9	32.9 ± 11.6	19.2 ± 2.9	20.1 ± 6.5	9.1 ± 7.1	326.0 ± 168.9	9.7 ± 3.9
*Cirsium canum*	7.1 ± 0.5	0.28 ± 0.06	3.3 ± 0.4	5.8 ± 0.7	13.4 ± 2.8	18.9 ± 2.2	11.4 ± 5.4	13.4 ± 2.7	12.2 ± 3.1	740.0 ± 314.3	12.6 ± 2.5
*Cnidum dubium*	5.5 ± 0.2	0.50 ± 0.02	5.3 ± 0.2	9.1 ± 0.3	10.6 ± 0.1	18.1 ± 2.0	18.1 ± 3.0	14.5 ± 1.3	15.8 ± 4.2	480.0 ± 153.9	16.3 ± 7.5
*Cucubalus baccifer*	7.7 ± 0.2	0.13 ± 0.07	1.7 ± 0.7	3.0 ± 1.2	14.0 ± 3.0	25.7 ± 14.7	12.2 ± 6.6	24.5 ± 13.6	6.4 ± 1.4	358.7 ± 119.7	10.0 ± 5.6
*Cyperus fusus*	6.8 ± 0.7	0.14 ± 0.12	1.9 ± 1.5	3.2 ± 2.6	14.2 ± 1.5	16.6 ± 13.0	12.8 ± 7.5	13.1 ± 11.2	10.2 ± 5.3	288.8 ± 151.3	6.0 ± 4.7
*Dipsacus laciniatus*	6.1 ± 0.3	0.15 ± 0.01	2.3 ± 0.2	4.0 ± 0.3	15.3 ± 0.4	26.4 ± 1.1	8.9 ± 0.1	17.8 ± 8.1	11.3 ± 1.1	335.0 ± 42.4	17.0 ± 0.7
*Equisetum ramosissimum*	7.3 ± 0.8	0.11 ± 0.08	1.3 ± 0.8	2.2 ± 1.4	13.1 ± 3.1	7.8 ± 1.7	7.4 ± 2.7	7.5 ± 2.2	4.5 ± 1.4	225.3 ± 45.7	4.0 ± 0.9
*Eryngium planum*	7.0 ± 0.7	0.12 ± 0.01	1.3 ± 0.2	2.3 ± 0.4	10.5 ± 0.8	19.0 ± 3.5	9.3 ± 2.6	12.1 ± 3.0	4.8 ± 2.8	248.0 ± 176.8	6.2 ± 2.4
*Gratiola officinalis*	7.1 ± 0.4	0.11 ± 0.03	1.6 ± 0.4	2.8 ± 0.7	15.5 ± 0.7	5.4 ± 0.6	6.2 ± 2.6	4.5 ± 0.7	4.8 ± 1.1	26.0 ± 14.1	1.3 ± 0.0
*Kochia laniflora*	6.2 ± 0.3	0.09 ± 0.00	1.0 ± 0.1	1.8 ± 0.1	10.9 ± 0.8	26.7 ± 2.1	11.2 ± 2.8	16.5 ± 1.7	1.7 ± 0.5	26.7 ± 4.2	1.3 ± 0.6
*Lavatera thuringiaca*	7.7 ± 0.2	0.10 ± 0.04	1.1 ± 0.5	1.9 ± 0.8	11.0 ± 0.0	46.3 ± 30.1	17.6 ± 10.4	26.4 ± 13.9	6.4 ± 2.5	289.3 ± 152.0	7.0 ± 1.3
*Leymus arenarius*	5.4 ± 0.6	0.11 ± 0.03	1.6 ± 0.7	2.8 ± 1.2	14.1 ± 3.3	5.3 ± 4.1	4.3 ± 2.2	5.3 ± 4.5	5.8 ± 5.5	16.3 ± 13.1	1.2 ± 1.6
*Limosella aquatica*	7.1 ± 0.2	0.08 ± 0.04	1.2 ± 0.7	2.1 ± 1.3	14.1 ± 3.2	23.2 ± 13.8	22.5 ± 16.5	16.7 ± 10.2	16.5 ± 11.8	323.7 ± 358.7	8.7 ± 9.9
*Lindernia procumbens*	6.1 ± 0.8	0.22 ± 0.07	3.4 ± 1.1	5.8 ± 1.9	15.1 ± 1.0	15.0 ± 5.6	14.1 ± 6.0	12.5 ± 6.6	11.0 ± 3.1	411.7 ± 45.4	9.0 ± 3.5
*Lithospermum officinale*	7.4 ± 0.0	0.12 ± 0.00	1.7 ± 0.0	3.0 ± 0.0	14.8 ± 0.2	11.9 ± 1.3	12.8 ± 0.3	10.3 ± 2.5	10.3 ± 0.4	320.0 ± 28.3	5.5 ± 2.1
*Myosotis sparsiflora*	7.4 ± 0.1	0.19 ± 0.04	3.0 ± 0.7	5.2 ± 1.2	15.8 ± 0.6	31.2 ± 6.2	58.5 ± 28.4	24.5 ± 0.7	8.8 ± 1.8	265.0 ± 75.0	4.0 ± 2.1
*Petasites spurius*	6.4 ± 0.3	0.07 ± 0.04	1.1 ± 0.6	1.8 ± 1.0	14.8 ± 2.5	3.2 ± 2.1	3.1 ± 2.4	3.0 ± 1.7	3.8 ± 2.4	47.7 ± 38.6	2.7 ± 1.9
*Potentilla supina*	6.9 ± 0.9	0.14 ± 0.12	2.0 ± 1.7	3.4 ± 2.9	14.0 ± 1.3	16.7 ± 17.5	12.5 ± 10.4	14.7 ± 15.1	10.2 ± 6.5	320.0 ± 217.9	4.7 ± 5.1
*Roripa austriaca*	7.6 ± 0.3	0.13 ± 0.02	1.7 ± 0.3	2.9 ± 0.6	12.6 ± 0.8	21.5 ± 14.0	22.1 ± 12.1	17.5 ± 9.8	6.0 ± 3.5	291.1 ± 210.1	2.5 ± 2.2
*Scutellaria hastifolia*	6.1 ± 0.3	0.27 ± 0.03	3.6 ± 0.6	6.3 ± 1.0	13.4 ± 1.3	12.0 ± 7.7	12.9 ± 10.3	11.7 ± 7.9	10.0 ± 2.6	299.0 ± 143.8	3.8 ± 4.6
*Senecio fluviatilis*	7.7 ± 0.2	0.19 ± 0.01	2.2 ± 0.1	3.7 ± 0.2	11.2 ± 0.1	20.9 ± 8.3	11.8 ± 4.1	16.2 ± 6.9	7.3 ± 1.2	548.7 ± 181.3	10. 5 ± 7.0
*Sisymbrium strictissimum*	7.8 ± 0.1	0.14 ± 0.06	1.8 ± 0.7	3.0 ± 1.1	13.2 ± 2.2	19.3 ± 8.3	12.6 ± 5.4	18.7 ± 7.4	8.5 ± 0.9	451.3 ± 88.6	8.3 ± 0.6
*Viola stagnina*	5.7 ± 0.3	0.27 ± 0.04	3.7 ± 0.2	6.4 ± 0.4	14.2 ± 1.9	16.9 ± 3.6	13.0 ± 11.8	15.3 ± 3.2	9.5 ± 2.3	159.3 ± 56.1	8.0 ± 1.8

Soil pH at sites where plant material was collected ranged from 5.4 (in the case of *Leymus arenarius*) to 7.8 (for samples from under *Sisymbrium strictissimum*). The highest pH was usually observed for tall-herbs typical of watercourse veils developing along the river. The lowest total nitrogen, organic carbon and organic matter contents were recorded for *Aethusa cynapioides*, whereas the highest values of these three parametres were observed in soils from under *Cnidium dubium*. Generally, meadow species grew on soil with the highest total nitrogen, organic carbon and organic matter contents in the study area. The C/N value ranged between 10.5 (for soil from under *Eryngium planum*) and 15.8 (for soil from under *Myosotis sparsiflora*). The lowest contents of plant-available phosphorus and potassium were observed for sandy soils (mostly soil from under *Leymus arenarius* and *Petasites spurius*) whereas the soils with the highest contents of the two substances were those collected from under the root systems of plants representing different types of habitats. Similar trends were also found for major exchangeable cations (K^+^, Na^+^, Mg^2+^ and Ca^2+^).

The Spearman’s rank correlation coefficients confirmed medium correlations between root colonization of RCP species by DSE and the contents of total nitrogen and organic carbon as well as soil organic matter (Table 3). Moreover, root colonization of RCP by *Olpidium* spp. correlated significantly with the plant-available potassium and Mg^2+^ contents, as well as pH value, but these correlations were rather weak. No statistically significant correlation between root colonization of RCP by AMF and soil parameters analyzed was found (Table 3).

## Discussion

In this paper, the detailed report on the occurrence of both AMF and fungal endophytes in roots of 33 plant species belonging to 18 families and confined to river corridors in Central Europe is presented. To our knowledge, the AM status of 26 plant species and seven genera is reported for the first time (see Table 2). The presence of AM in *Allium scorodoprasum* and *Gratiola officinalis* is consistent with the literature data. *Butomus umbelatus* and *Lithospermum officinale*, being reported earlier as non-mycorrhizal, were recognized in our studies as colonized by AMF. The roots of *Bolboschoenus maritimus* were devoid of AMF, contrary to previous reports. *Alisma lanceolatum* and *Equisetum ramosissimum* were also found not to form AM whereas, in earlier research, these species were observed to be both non-mycorrhizal and associated with AMF (Dickson et al. 2007; Wang and Qiu 2006; Zubek et al. 2011a).

Nineteen of the studied plant species formed AM associations. AMF mycelium was not found in the root systems of 14 plants. Most of these non-mycorrhizal species belong to Brassicaceae, Caryophyllaceae, Chenopodiaceae and Cyperaceae, that are generally accepted as conventionally AMF non-host families (Smith and Read 2008; Wang and Qiu 2006). In addition, *Alisma lanceolatum* (Alismataceae), *Leymus arenarius* (Poaceae), *Equisetum ramosissimum* (Equisetaceae), *Myosotis sparsiflora* (Boraginaceae) and *Limosella aquatica* (Scrophulariaceae) were also found not to form AM symbiosis. This may be due to the lack of AMF propagules in the sites of these plants. This seems to be especially the case for *Leymus arenarius*, *Equisetum ramosissimum* and *Limosella aquatica* as in the trap cultures established with soils collected from under the root systems of these plants no AMF spores were found. However, in the case of *Alisma lanceolatum* and *Myosotis sparsiflora* the spores were found in the trap cultures. This might indicate that the absence of AMF propagules in the soil was not the reason for the lack of mycorrhizae. The first possible explanation for the absence of AM in the two species may be the lack of functional need for mycorrhizal association in these particular edaphic conditions, e.g. due to sufficient soil nutrient contents. This supposition seems to be confirmed mostly by the chemical properties of soils from under *Myosotis* *sparsiflora* (Table 4), however, the statistical analysis performed indicated no significant correlation between root colonization of RCP species by AMF and particular soil parameters. On the other hand, there is increasing evidence for some degree of physical and functional specificity in the symbiosis (Smith and Read 2008; Wubet et al. 2006). Therefore, if some AMF species are required for particular plants, the lack of compatible fungal symbionts in the soil may also be a reason for the absence of root colonization. Such a situation might occur, in particular, in river corridor habitats such as those characterized by rather low AMF species diversity, as it was shown in our studies (Table 1). In the studies by Błaszkowski et al. (2002); Zubek et al. (2008) and Zubek et al. (2009) on AMF species richness conducted in more stable plant communities in Southern Poland, 22, 20 and 15 AMF species, respectively, were found. Moreover, Kivlin et al. (2011), based on a synthesis of published DNA sequences, revealed that natural ecosystems harbor, on average, 15–30 AMF species.

AMF species found in the present study commonly occur in Poland and also have wide distribution in the world (Błaszkowski 2012; Błaszkowski et al. 2012). Although AMF spores were found in only 32 % of the trap cultures established with the soils collected from under ten plant species, the AMF propagules were present in more sites as mycorrhizal colonization was found in the roots of 19 plant species. The establishment of trap cultures frequently stimulates sporulation of AMF and reveals species that produce spores either seasonally or not at all in natural habitats (Stutz and Morton 1996). Nevertheless, some AMF may, in contrast, not grow or produce spores in a laboratory probably as a result of high ecological (environmental) differences which may be owing to either edaphic conditions or the aforementioned AMF–host plant specificity (Zubek et al. 2013).

Floods typical of river corridors may cause significant reduction of gas exchange especially underground and can promote soil erosion. These usually result in decrease in survival or establishment of many plant species causing simultaneous reduction of plant cover, but it can also influence soil microorganisms, including AMF. Variable soil moisture in river valleys is undoubtedly also important for the occurrence of different organisms (e.g. Hensgen et al. 2011). We observed the presence of AMF in the majority of river valley sites under study, as revealed by both the observation of fungi colonizing roots and the presence of their spores in the trap cultures. Our result is in line with literature data, which confirms the adaptation of AMF to harsh conditions. The presence of AMF has been observed in wet habitats (Ingham and Wilson 1999; Kołaczek et al. 2013; Weishampel and Bedfor 2006). The colonization of roots was found even in plants experiencing flooded conditions (Bohrer et al. 2004; Miller 2000; Stevens et al. 2011). The presence of AMF communities has been also found in diverse habitats exposed to extreme drought stress (Al-Yahya’ei et al. 2011; Chaudhry et al. 2005). Furthermore, it was shown that mechanical soil disturbance reduced AMF propagule abundance (Gosling et al. 2006; Kabir 2005), spore numbers (Galvez et al. 2001) and species/phylotype richness (Brito et al. 2012; Schnoor et al. 2011). Although Jansa et al. (2002, 2003) found that soil disturbance had generally negative effect on the abundance of some AMF species, relatively high AMF diversity was still detected in disturbed soils. Some AMF species were more tolerant to the disturbance due to differences in life strategies among fungal taxa, namely disparate growth patterns and propagules (Schnoor et al. 2011).

The mycelia of dark septate endophytes (DSE) were found in 15 plants. DSE co-occurred with AMF in the roots of several species, but also occurred in plants that were non-mycorrhizal. DSE represent a taxonomically diverse group of fungi. They are frequently encountered root-inhabiting fungi of numerous plant species from a wide range of terrestrial ecosystems (Newsham 2011; Weishampel and Bedfor 2006). The effects of these endophytes on plants are diverse and may depend on the host species, fungal taxa or strains, and environmental conditions (Andrade-Linares et al. 2011; Mandyam and Jumpponen 2005; Mandyam et al. 2012; Newsham 2011; Wu et al. 2010). In addition to the presence of AMF and DSE mycelia in the roots of RCP under study, the sporangia of *Olpidium* spp. were found sporadically in ten species. The taxonomic position of this group of fungi, traditionally placed in Chytridiomycota, is currently considered to be uncertain (Hibbett et al. 2007). Nevertheless, their biology has been well-documented. Several species from the *Olpidium* genus were found to be symptomless root parasites and also the vectors of plant viruses (Webster and Weber 2007; Verchot-Lubicz 2003). The correlation between root colonization of RCP species by DSE and *Olpidium* spp. and some soil chemical properties is not apparent and requires further investigations.

In conclusion, the obtained results only to some extent confirm our supposition that species confined to river corridors do not form AM or form it rarely and that the degree of colonization of their roots is low due to the lack of AMF propagules in their habitats. AMF are believed to colonize roots of the great majority (ca. 80 %) of vascular plants in the world (Perotto et al. 2013; Smith and Read 2008). Our study of 33 RCP species indicated that 14 of the examined species were non-mycorrhizal. Although 19 species were mycorrhizal and most of them were characterized by high mycorrhizal frequency and also colonization rate, the possibility that some of them are not dependent on AMF and do not benefit from this symbiotic association in stable plant-AMF communities outside river corridors can not be excluded. It is worth noting that research carried out by Donath et al. (2003) showed that in the case of some RCP favorable site conditions do not guarantee their restoration success. What is more, in the experimental studies, Fischer et al. (2010) showed that species more confined to river corridor areas are less able to take advantage of more benign conditions (i.e., absence of flooding and more favorable soil properties) typical for non-river corridor habitats than the species of wide distributional range.

Among 85 vascular plant species which are considered to be confined to river corridors in Poland (Nobis and Skórka 2014), but not studied during our research, there are some which are not associated with AMF because they are parasitic plants (e.g. *Cuscuta lupuliformis*) or have no roots (e.g. *Salvinia natans*, *Wolffia arrhiza*). Many other (e.g. *Arabis planisiliqua*, *Cardamine parviflora*, *Cerasitum dubium*, *Chenopodium acerifolium*, *Corrigiola litoralis*, *Dichostylis mischeliana*, *Erysimum hieracifolium*, *Rumex paluster*, *Rumex ucrainicus*, *Silene tatarica*) with high probability are non-mycorrhizal as they represent families or genera that are generally considered as non-mycorrizal (Smith and Read 2008; Wang and Qiu 2006). Therefore, the final percentage of non-mycorrhizal species in the group of RCP may be large and not accidental.

It must be emphasized that for a particular river corridor species its corridor-like distribution can be determined by the combination of certain abiotic and biotic variables, but because the group of plants in question is very heterogenous, it is unlikely that a single hypothesis could explain the river corridor distribution pattern (Burkart 2001). It is known that some non-mycorrhizal plants may have significantly lower competitive ability in comparison with those which are mycorrhizal (Smith and Read 2008). Therefore, it is probable that the river corridor species under study which turned out to be non-mycorrhizal are simply outcompeted outside river valleys. This supposition, however, requires confirmation. Further studies (including experimental) on the role of AMF and other endophytes in RCP distribution are thus needed.

## Electronic supplementary material

Below is the link to the electronic supplementary material.

Supplementary material 1 (TIFF 20749 kb)

Supplementary material 2 (DOC 167 kb)
