# Supplementary figures and images for: Arbuscular mycorrhizal fungi associations of vascular plants confined to river valleys: towards understanding the river corridor plant distribution
Source: J Plant Res. 2014 Nov 25;128(1):127–37. doi: 10.1007/s10265-014-0680-9 (PMC4302419; doi:10.1007/s10265-014-0680-9)

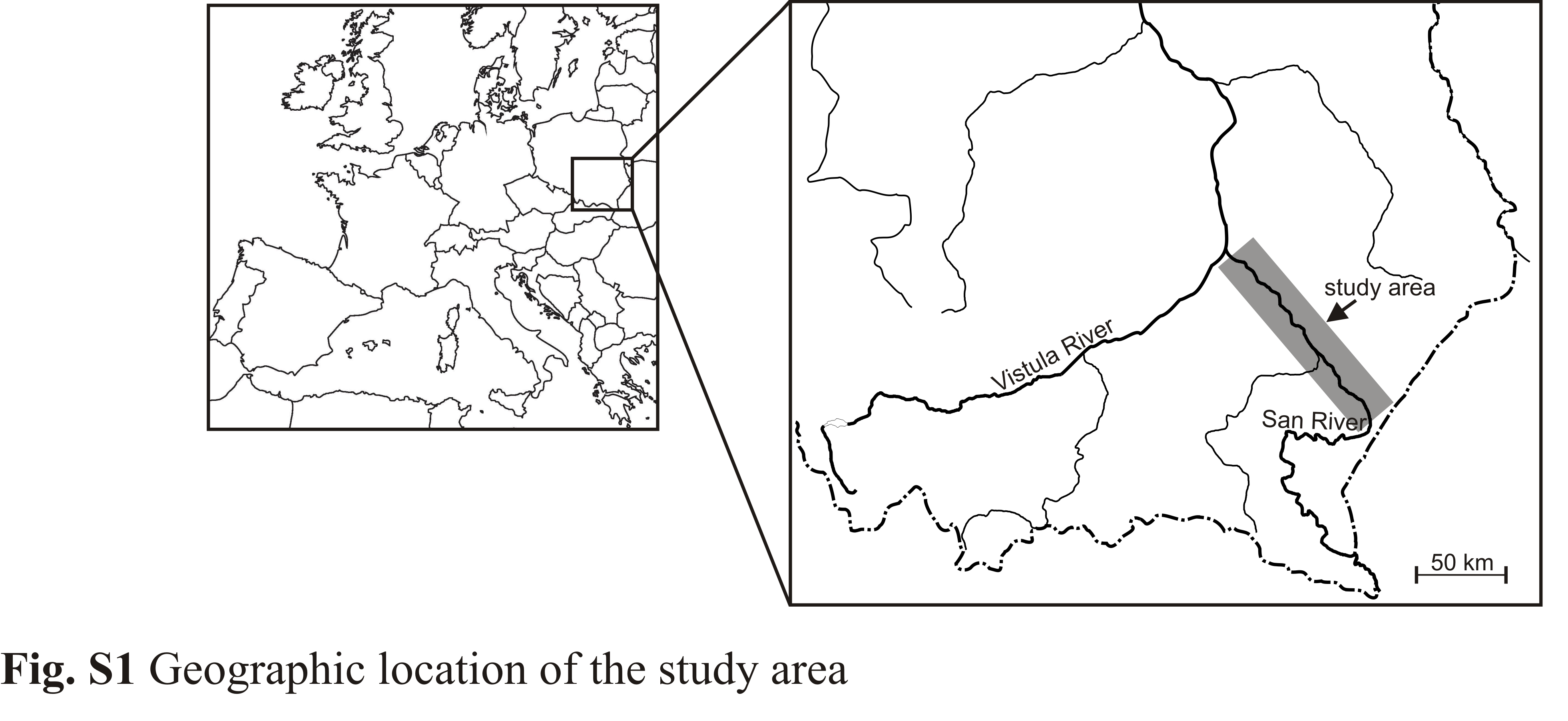

Supplement: Supplementary file 1 — Supplementary material 1 (TIFF 20749 kb) [file 10265_2014_680_MOESM1_ESM.tif]
