# Supplementary material for: Arbuscular mycorrhizal fungi associations of vascular plants confined to river valleys: towards understanding the river corridor plant distribution
Source: J Plant Res. 2014 Nov 25;128(1):127–37. doi: 10.1007/s10265-014-0680-9 (PMC4302419; doi:10.1007/s10265-014-0680-9)
Supplement: Supplementary file 2 — Supplementary material 2 (DOC 167 kb) [file 10265_2014_680_MOESM2_ESM.doc]

**Table S1** A list of the studied river corridor species together with the data on the name and coordinates of localities as well as the habitats where their specimens were collected

| Vascular plant species | Localization ^a^ | Coordinates ^b^ | | Habitat |
| --- | --- | --- | --- | --- |
| Aethusa cynapioides | Stare Miasto **^1^** | N 50° 18’ 58.0” | E 22° 25’ 42.8” | watercourse veils along the San River |
|  | Krzeszów **^2^** | N 50° 24’ 07.8” | E 22° 20’ 05.5” | watercourse veils along the San River |
|  | Jarosław **^3^** | N 50° 01’ 14.0” | E 22° 42’ 13.6” | watercourse veils along the San River |
| Alisma lanceolatum | Ostrów | N 49° 56’ 53.4” | E 22° 46’ 36.4” | swamp |
|  | Popówka | N 50° 21’ 55.0” | E 22° 22’ 58.2” | swamp |
|  | Kępa **^4^** | N 50° 39’ 37.1” | E 21° 55’ 56.9” | wet arable field |
| *Allium angulosum* | Jarosław | N 50° 02’ 37.3” | E 22° 40’ 02.7” | wet meadow |
|  | Rzuchów | N 50° 15’ 28.5” | E 22° 30’ 15.7” | wet meadow |
|  | Piskorowice | N 50° 13’ 07.3” | E 22° 30’ 53.3” | wet meadow |
| *Allium scorodoprasum* | Radymno | N 49° 57’ 44.3” | E 22° 48’ 35.5” | wet meadow |
|  | Jarosław | N 50° 03’ 10.1” | E 22° 41’ 11.0” | freash meadow |
|  | Jarosław | N 50° 02’ 56.4” | E 22° 41’ 19.7” | freash meadow |
| Barbarea stricta | Krzeszów **^5^** | N 50° 24’ 03.5” | E 22° 20’ 03.3” | watercourse veils along the San River |
|  | Rzuchów **^6^** | N 50° 14’ 01.2” | E 22° 30’ 08.3” | watercourse veils along the San River |
|  | Chłopska Wola **^7^** | N 50° 35’ 43.4” | E 22° 04’ 10.8” | watercourse veils along the Bukowa River |
| *Bolboschoenus maritimus* | Piskorowice | N 50° 14’ 08.8” | E 22° 30’ 27.9” | oxbow lake |
|  | Ostrów | N 49° 56’ 55.6” | E 22° 46’ 36.3” | flooded arable field |
|  | Radymno | N 49° 57’ 04.9” | E 22° 49’ 46.6” | bank of an artificial water reservoir |
|  | Radymno | N 49° 57’ 07.4” | E 22° 49’ 49.0” | flooded bank of an artificial water reservoir |
| Butomus umbelatus | Kępa **^8^** | N 50° 39’ 37.1” | E 21° 55’ 56.9” | wet arable field |
|  | Grabczyny **^9^** | N 50° 39’ 41.0” | E 21° 56’ 09.2” | bank of an oxbow lake |
|  | Leżachów **^10^** | N 50° 09’ 01.4” | E 22° 37’ 14.1” | bank of a pond |
| Carex praecox | Chłopska Wola **^11^** | N 50° 35’ 44.2” | E 22° 04’ 11.1” | dry grassland |
|  | Piwoda **^12^** | N 50° 03’ 56.1” | E 22° 47’ 02.3” | fresh meadow |
|  | Leżachów **^13^** | N 50° 08’ 40.0” | E 22° 36’ 10.6” | fresh meadow |
|  | Ożanna Mała **^14^** | N 50° 17’ 26.0” | E 22° 29’ 01.3” | fresh meadow |
| *Chaerophyllum bulbosum* | Jarosław | N 50° 03’ 14.9” | E 22° 41’ 43.7” | watercourse veils along the San River |
|  | Krzeszów | N 50° 24’ 07.8” | E 22° 20’ 05.5” | watercourse veils along the San River |
|  | Rzuchów | N 50° 14’ 50.4” | E 22° 30’ 06.5” | watercourse veils along the San River |
|  | Sieniawa | N 50° 09’ 09.0” | E 22° 35’ 10.5” | watercourse veils along the San River |
| Chaiturus marrubiastrum | Leżachów **^15^** | N 50° 09’ 01.2” | E 22° 37’ 08.7” | swamp |
|  | Leżachów **^16^** | N 50° 09’ 01.0” | E 22° 36’ 59.2” | drainage ditch |
|  | Leżachów **^17^** | N 50° 09’ 01.0” | E 22° 36’ 54.6” | drainage ditch |
| *Chenopodium ficifolium* | Kuryłówka | N 50° 17’ 28.1” | E 22° 26’ 27.4” | watercourse veils along the San River |
|  | Piskorowice | N 50° 13’ 45.1” | E 22° 30’ 28.3” | bank of the San River |
|  | Piskorowice | N 50° 13’ 38.6” | E 22° 30’ 40.0” | bank of the San River |
|  | Radymno | N 49° 57’ 31.6” | E 22° 48’ 26.5” | wasteland |
| Cirsium canum | Radymno | N 49° 57’ 26.7” | E 22° 48’ 21.4” | drainage ditch |
|  | Radymno | N 49° 57’ 44.3” | E 22° 48’ 35.5” | wet meadow |
|  | Jarosław **^18^** | N 50° 02’ 37.3” | E 22° 40’ 02.7” | wet meadow |
| Cnidum dubium | Krzeszów Dolny **^19^** | N 50° 23’ 07.5” | E 22° 21’ 59.1” | wet meadow |
|  | Krzeszów Dolny **^20^** | N 50° 23’ 00.0” | E 22° 21’ 35.9” | fresh meadow |
|  | Jarosław **^21^** | N 50° 02’ 37.3” | E 22° 40’ 02.7” | wet meadow |
| Cucubalus baccifer | Walawa | N 49° 51’ 33.1” | E 22° 53’ 20.5” | watercourse veils along the San River |
|  | Jarosław | N 50° 03’ 05.0” | E 22° 41’ 49.8” | watercourse veils along the San River |
|  | Sieniawa | N 50° 09’ 59.5” | E 22° 35’ 09.5” | watercourse veils along the San River |
| Cyperus fusus | Zbydniów **^22^** | N 50° 38’ 27.5” | E 21° 54’ 48.4” | exposed pond bottom |
|  | Grabczyny **^23^** | N 50° 39’ 41.0” | E 21° 56’ 09.2” | shore of an oxbow lake |
|  | Grabczyny **^24^** | N 50° 39’ 51.4” | E 21° 56’ 42.3” | shore of an oxbow lake |
|  | Kuryłówka **^25^** | N 50° 18’ 41.2” | E 22° 25’ 58.1” | bank of the San River |
| Dipsacus laciniatus | Leżachów **^26^** | N 50° 09’ 01.0” | E 22° 36’ 55.8” | fresh meadow |
|  | Leżachów **^27^** | N 50° 09’ 01.5” | E 22° 36’ 53.6” | drainage ditch |
| *Equisetum ramosissimum* | Kuryłówka | N 50° 17’ 27.0” | E 22° 26’ 41.8” | wasteland on sandy soil |
|  | Nowa Wieś | N 50° 30’ 33.4” | E 22° 12’ 13.0” | grassland on loose sandy soil |
|  | Krzeszów | N 50° 24’ 03.9” | E 22° 20’ 07.0” | wet grassland on sandy soil |
| *Eryngium planum* | Sieniawa | N 50° 10’ 00.3” | E 22° 35’ 12.5” | roadside |
|  | Rzuchów | N 50° 14’ 52.4” | E 22° 30’ 09.6” | fresh meadow |
|  | Wierzawice | N 50° 13’ 00.1” | E 22° 28’ 22.6” | fresh meadow |
| Gratiola officinalis | Grądy **^28^** | N 50° 29’ 41.2” | E 22° 09’ 18.6” | drainage ditch along a road |
|  | Grądy **^29^** | N 50° 29’ 36.7” | E 22° 09’ 10.6” | wasteland by the Barcówka watercourse |
| *Kochia laniflora* | Nowa Wieś | N 50° 30’ 33.4” | E 22° 12’ 13.0” | dry grassland on loose sandy soil |
|  | Nowa Wieś | N 50° 30’ 39.1” | E 22° 12’ 14.0” | dry grassland on loose sandy soil |
|  | Krzeszów Dolny | N 50° 23’ 17.7” | E 22° 21’ 58.3” | dry grassland on loose sandy soil |
| *Lavatera thuringiaca* | Nowa Wieś | N 50° 30’ 49.6” | E 22° 11’ 59.6” | herbaceous fringes |
|  | Walawa | N 49° 51’ 35.4” | E 22° 53’ 21.1” | watercourse veils along the San River |
|  | Jarosław | N 50° 03’ 11.6” | E 22° 41’ 45.9” | watercourse veils along the San River |
| Leymus arenarius | Kamionka **^30^** | N 50° 25’ 3.9” | E 22° 19’ 20.3” | dry grassland on loose sandy soil |
|  | Krzeszów Dolny **^31^** | N 50° 23’ 03.2” | E 22° 21’ 57.7” | dry grassland on loose sandy soil |
|  | Krzeszów Dolny **^32^** | N 50° 23’ 12.2” | E 22° 21’ 51.0” | dry grassland on loose sandy soil |
| Limosella aquatica | Piskorowice | N 50° 13’ 35.7” | E 22° 30’ 45.7” | muddy bank of the San River |
|  | Zbydniów **^33^** | N 50° 38’ 33.5” | E 21° 54’ 48.2” | exposed pond bottom |
|  | Zbydniów **^34^** | N 50° 38’ 27.5” | E 21° 54’ 48.4” | exposed pond bottom |
| Lindernia procumbens | Rzuchów **^35^** | N 50° 15' 21.6” | E 22° 29’ 39.3” | wet arable field (cultivation of *Zea mays*) |
|  | Kuryłówka **^36^** | N 50° 18’ 01.4” | E 22° 27’ 08.2” | muddy bank of the Złota River |
|  | Kuryłówka **^37^** | N 50° 17’ 07.1” | E 22° 27’ 16.4” | muddy bank of the Złota River |
| Lithospermum officinale | Grabczyny **^38^** | N 50° 39’ 55.9” | E 21° 56’ 59.5” | dry grassland |
|  | Grabczyny **^39^** | N 50° 39’ 57.8” | E 21° 56’ 54.5” | dry grassland |
| Myosotis sparsiflora | Walawa **^40^** | N 49° 51’ 43.3” | E 22° 53’ 29.9” | riverine scrub along the San River |
|  | Walawa **^41^** | N 49° 51’ 32.7” | E 22° 53’ 20.5” | riverine scrub along the San River |
| Petasites spurius | Ulanów **^42^** | N 50° 30’ 06.5” | E 22° 16’ 07.4” | dry grassland on sandy soil |
|  | Ulanów **^43^** | N 50° 29’ 06.5” | E 22° 16’ 58.1” | dry grassland on sandy soil |
|  | Ulanów **^44^** | N 50° 29’ 54.8” | E 22° 16’ 10.4” | dry grassland on sandy soil |
| Potentilla supina | Leżachów **^45^** | N 50° 09’ 00.9” | E 22° 37’ 07.4” | drainage ditch |
|  | Zbydniów **^46^** | N 50° 38’ 33.5” | E 21° 54’ 48.2” | shore of an oxbow lake |
|  | Grabczyny **^47^** | N 50° 39’ 41.0” | E 21° 56’ 09.2” | shore of an oxbow lake |
| Roripa austriaca | Rzuchów **^48^** | N 50° 14’ 52.4” | E 22° 30’ 09.6” | fresh meadow |
|  | Krzeszów **^49^** | N 50° 24’ 05.3” | E 22° 20’ 04.5” | fresh meadow |
|  | Krzeszów **^50^** | N 50° 24’ 02.0” | E 22° 20’ 07.9” | roadside |
| Scutellaria hastifolia | Racławice **^51^** | N 50° 30’ 32.3” | E 22° 10’ 40.2” | fresh meadow |
|  | Racławice **^52^** | N 50° 30’ 28.9” | E 22° 10’ 40.1” | wet meadow |
|  | Wierzawice **^53^** | N 50° 13’ 14.4” | E 22° 29’ 33.9” | drainage ditch |
| *Senecio fluviatilis* | Łazów | N 50° 20’ 09.9” | E 22° 24’ 54.2” | watercourse veils along the San River |
|  | Krzeszów | N 50° 24’ 01.6” | E 22° 20’ 03.3” | watercourse veils along the San River |
|  | Sieniawa | N 50° 09’ 58.1” | E 22° 35’ 12.0” | watercourse veils along the San River |
| *Sisymbrium strictissimum* | Sieniawa | N 50° 09’ 09.0” | E 22° 35’ 10.5” | watercourse veils along the San River |
|  | Rzuchów | N 50° 15’ 10.4” | E 22° 29’ 27.9” | watercourse veils along the San River |
|  | Jarosław | N 50° 03’ 17.0” | E 22° 41’ 42.4” | watercourse veils along the San River |
| Viola stagnina | Leżachów **^54^** | N 50° 09’ 01.1” | E 22° 36’ 50.9” | drainage ditch |
|  | Rzuchów **^55^** | N 50° 15’ 15.1” | E 22° 29’ 39.0” | drainage ditch |
|  | Rzuchów **^56^** | N 50° 15’ 20.7” | E 22° 29’ 45.2” | wet meadow |

^a^ The number after the location indicates the trap culture

^b^ The location was determined using GPS receiver (the WGS84 coordinate system)
